# A mixed methods exploration of patterns of healthcare utilization of urban women with non-communicable disease in South Africa

**DOI:** 10.1186/s12913-014-0528-y

**Published:** 2014-11-04

**Authors:** Daniel Lopes Ibanez-Gonzalez, Emily Mendenhall, Shane A Norris

**Affiliations:** MRC/Wits Developmental Pathways for Health Research Unit, University of the Witwatersrand, Johannesburg, South Africa; Walsh School of Foreign Service, Georgetown University, Washington, DC USA

**Keywords:** Healthcare utilization, Non-communicable disease, Mixed methods, Trust

## Abstract

**Background:**

Despite the growing burden of NCDs in South Africa, very little is known about how people living in urban townships manage these illnesses. In this article we expound upon the findings of a study showing that only one-third of women with an NCD participating in the Birth to Twenty (Bt20) cohort study of Soweto-Johannesburg, South Africa, had sought biomedical services in the previous six months.

**Methods:**

We evaluated quantitative data from a cross sectional health access survey conducted with adult women (mean age = 44.8) and examined 25 in-depth narrative interviews with twelve women who self-reported at least one NCD from the larger study.

**Results:**

The qualitative findings highlight the potential role of negative experiences of healthcare services and biomedicine in delaying the seeking of healthcare. Multivariate analysis of the quantitative findings found that the possession of medical aid (OR = 1.7, CI = 1.01-2.84) and the self-reported use of patient strategies in negotiating healthcare access (OR = 1.6, CI = 1.04-2.34) were positively associated with the utilization of healthcare services. Belief in the superior efficacy of traditional healers over doctors was associated with delay of NCD treatment (OR = 2.4, CI = 1.14-4.18).

**Conclusion:**

Our data suggest that low healthcare utilization is due in part to low rates of expectation for consistent and high-quality care and potential mistrust of the medical system. We conclude that both demand-side and supply-side measures focusing on high trust management practices will prove essential in ensuring access to healthcare services.

**Electronic supplementary material:**

The online version of this article (doi:10.1186/s12913-014-0528-y) contains supplementary material, which is available to authorized users.

## Background

Non-communicable diseases (NCDs), such as hypertension and type-2 diabetes, are the leading causes of death globally, contributing to two-thirds of global mortality [1]. Around 80 percent of these deaths occur in low- and middle-income countries (LMICs). Although historically NCDs afflicted affluent populations, increases in NCD prevalence, morbidity, and mortality have been measured among lower income groups [2,3]. This is largely due to increases in obesity that have corresponded with the increased availability and consumption of higher caloric diets, lower physical activity due to mechanization, and tobacco use [2,3]. As these lifestyle trends move from the affluent to the middle class and working poor, increasing NCD incidence and prevalence confront inadequate healthcare services for these populations in many LMICs [4].

Evidence from South Africa suggests that increasing NCDs coexist with low healthcare utilization [5]. For example, the South Africa Health and Demographic Survey found affordability and availability of healthcare services prevented 39% and 31% of women, respectively, from accessing healthcare in 2003 [6]. Some of the healthcare access issues may result from a large burden of disease upon a healthcare system historically rendered inequitable and inefficient by a racially-fragmented public healthcare approach [7]. One of the greatest challenges for NCD care involves universal coverage of healthcare services, which integrate community-based preventive services with hospital-based curative care [8].

Despite the growing burden of NCDs in South Africa, very little is known about how people living in urban townships manage these illnesses and engage with biomedical healthcare systems. We found in a healthcare access survey conducted with late-adult women in Soweto that around 50% had been diagnosed with an NCD, from hypertension to diabetes and epilepsy. However, only one-third (33.3%) of these women had sought biomedical services for an illness episode in the past six months [9]. In our initial analysis, we could not attribute the low utilization of healthcare services either to problems with the availability or affordability of healthcare services, which was relatively high, nor the professed belief in traditional healers, since this appeared to be related primarily to problems not associated with NCDs or conditions understood as biomedical in nature. Rather, we explained these findings with reference to the fact that they indicate partial compliance with medical regimens in accordance with largely self-defined treatment plans [9,10].

Whereas we have briefly described patterns of healthcare utilization in Soweto, the aim of the present article is to further explore the reasons for these findings by the consideration of additional data collected in the health access survey, in a supplementary qualitative sub-study, and with reference to Andersen and Newman’s framework of healthcare utilization.

Andersen and Newman’s framework views healthcare utilization as an individual behaviour which is influenced by societal determinants, both directly and through the healthcare system [11]. As applied to the current study, we are interested in exploring the factors facilitating or inhibiting formal healthcare utilization defined as the multiple use of formal healthcare services over a period of time for the treatment of NCDs. Andersen and Newman speculate that in this type of scenario, societal level determinants, consisting primarily of technology and norms, may prove decisive [11].

## Methods

This study was conducted with the female caregivers (mothers) of the Birth to Twenty (Bt20) cohort, based in Soweto-Johannesburg, South Africa. The Bt20 cohort began in 1989/1990 as a longitudinal study of children’s health and wellbeing [12]. A total of 3 273 singleton children born between April and June 1990 and residing in Soweto-Johannesburg were enrolled in the study. [13,14]. The Bt20 cohort is currently in its 24th year, and has completed 20 data collection waves. The study is in contact with 70% of the initial cohort [9].

### Data source

The quantitative data comes from a cross sectional health access survey conducted with the primary Bt20 caregivers residing in Soweto. The study instrument combined adapted elements from the Adult Questionnaire of the South African Demographic and Health Survey [6] with standard community and demographic measures employed in the Bt20 study. The questionnaire included measures of availability and affordability of healthcare services as well as other domains [9]. With regard to healthcare access, we asked participants whether specific healthcare services (including private and public doctors, clinics and hospitals, as well as traditional healers, herbalists and social services) were available within a two kilometre radius of their homes, and, if so, whether they considered these services to be affordable for them. The questionnaire was piloted before being administered from November 2008 to June 2010. The questionnaire was administered in the homes of the study participants in their home language by a team of research assistants.

In addition to the survey, we conducted 25 in-depth narrative interviews with 12 caregivers who self-reported at least one NCD and the use of at least one support system in the health access survey. The interview guide was designed to provide insight into beliefs regarding health and healthcare access. It included 3 themes, along with subsidiary questions regarding: (i) initial responses to NCDs; (ii) experiences with formal healthcare services; and (iii) coping with NCDs.

The qualitative study participants were randomly selected and had one or more NCDs. Each participant was interviewed twice during the period of October 2009 to February 2010. The interviews were transcribed and translated in the same document, and the accuracy of the translations was checked by a research assistant not involved with data collection. The study protocol was approved by the Human Research Ethics Committee (Medical) of the University of the Witwatersrand (M090235), and pseudonyms are used in reporting the qualitative findings.

### Quantitative data analysis

In the present analysis we developed a series of tables describing sample characteristics, including sociodemographics and NCD characteristics including type of NCD, duration of illness and comorbidity. In contrast to our previous analysis [9], the current analysis excludes respondents who reported having tuberculosis, thus keeping the focus on participants with NCDs.

Drawing upon the behavioral model of Andersen and Newman, we conducted a series of Pearson’s Chi-squared analyses to determine potential relationships between NCD status and individual, societal, and healthcare system characteristics. Similar tables were developed using healthcare utilization as a dependent variable.

Whereas the two-way tables present potential relationships between individual, societal and healthcare system characteristics and healthcare utilization, multivariate logistic regression was required to show the adjusted effects of the combined determinants on healthcare utilization as our primary outcome of concern. In addition, we also explored type of healthcare utilization, and delay in formal treatment for NCDs as additional outcomes of interest.

Individual determinants included the possession of medical aid, age, employment status, socioeconomic status and the use of lifelong medication for the treatment of NCDs. Societal level determinants focused on norms such as belief in the efficacy of traditional healers as opposed to formal healthcare services, shared healthcare beliefs with community, and the use of patient strategies. The use of patient strategies was determined by the combination of two statistically associated Likert-scale measures regarding the selective disclosure of the symptoms of illness, and the private rehearsal of what symptoms to present to formal healthcare workers. Healthcare system determinants included the availability and affordability of formal healthcare services.

### Qualitative data analysis

Qualitative data analysis proceeded in a multi-staged process. In the first stage immediately following the initial interview key themes were identified and summarized in field notes. These themes served the dual purpose of prompting further development of NCD and healthcare service narratives in the follow-up interviews and the development of an initial coding schema for ongoing data analysis. These themes would be further elaborated in the final version of the interview fieldnotes, following the second interview.

In the second stage of data analysis the initial coding schema was further developed along-side an initial review of the translated interview transcripts in an iterative process, whereby coding of the transcripts and the development of the coding schema complemented each other. The final coding schema together with sample interview transcripts was reviewed by an independent researcher not connected with the study. In the final stage, the coded portions of the interview were summarized. Exemplar case studies of the general themes are presented in this paper to highlight common themes which complement the quantitative findings.

## Results

Descriptions of the demographic and NCD characteristics of the sample are provided in Tables 1 and 2. Half (50.2%, *n* = 547) the survey participants reported having been previously diagnosed with one or more of the following NCDs (Table 1): heart attack or angina, stroke, high blood cholesterol, diabetes, emphysema, asthma, arthritis, osteoporosis, epilepsy, or cancer. High blood pressure was the most prevalent form of NCD, affecting 32.8% of the study participants, followed by arthritis, which affected 12.9% of the population and high blood cholesterol, which affected 8.8% of the population (Table 2). The average duration of any NCD was 8.5 years (Table 1), and older participants were more likely to report one or more NCDs than younger study participants (Table 3). We found no relationship between socio-economic status and being diagnosed with an NCD, but those who were unemployed were more likely to report a NCD than those who were employed (Table 3).

**Table 1:** **Sample characteristics**

	**N (%)**
Age (N = 1089)	
30-39 yrs	263 (24.2%)
40-49 yrs	582 (53.4%)
50-65 yrs	244 (22.4%)
Socioeconomic status (N = 1086)	
Low	187 (17.2%)
Middle	490 (45.1%)
High	409 (37.7%)
Employment (N = 1090)	
Formal or informal paid labor	513 (47.1%)
Housewife/pensioner/unemployed	577 (52.9%)
NCD status (N = 1090)	
None reported	543 (49.8%)
One NCD reported	328 (30.1%)
More than one NCD reported	219 (20.1%)
Lifelong Medication (N = 547)	381 (69.7%)
NCD duration (years) (N = 452)	8.5 ± 9.2
Treatment duration (years) (N = 405)	8.1 ± 9.2
Delay in treatment (years) (N = 405)	0.8 ± 3.1

**Table 2:** **NCD prevalences** (**N** = **547**)

**NCD**	**N** **(responses)**	**% responses**	**% cases**
High blood pressure	358	43%	32.8%
Arthritis	141	16.9%	12.9%
High blood cholesterol	96	11.5%	8.8%
Diabetes	71	8.5%	6.5%
Asthma	54	6.5%	5%
Emphysema/bronchitis	33	4%	3%
Heart attack or angina	30	3.6%	2.8%
Osteoporosis	20	2.4%	1.8%
Stroke	18	2.2%	1.7%
Epilepsy	7	0.8%	0.6%
Cancer	4	0.5%	0.4%
Total	832	100%	76.3%

**Table 3:** **Correlating NCD status with individual**, **societal and healthcare system determinants and healthcare utilization**

**Domain**	**Determinants**	**No NCD**	**NCD**	**Total**	**P-** **value**
**Individual determinants**	Age (mean ± SD) (N = 1089)	43.7 ± 5.8	45.7 ± 6.6	44.7 ± 6.3	p = 0.00
	Socioeconomic Status (N = 1085)				
	Low	102 (18.9%)	85 (15.6%)	187 (17.2%)	p = 0.16
	Medium to High	439 (81.2%)	459 (84.4%)	898 (82.8%)	
	Employment (N = 1089)				
	Formal or informal paid labor	287 (52.9%)	225 (41.1%)	512 (47%)	p = 0.00
	Housewife/pensioner/unemployed	255 (47.1%)	322 (58.8%)	577 (53%)	
	Medical aid (N = 1086)	98 (18.2%)	94 (17.2%)	894 (82.3%)	p = 0.67
**Societal determinants**	Shared hlth beliefs family (N = 1090)	540 (99.5%)	542 (99.1%)	1082 (99.3%)	p = 0.48
	Shared hlth beliefs comm (N = 1090)	435 (80.1%)	446 (81.5%)	881 (80.8%)	p = 0.5
	Specific belief in traditional healers (N = 1085)	170 (31.4%)	175 (32.2%)	345 (31.8%)	p = 0.79
	Use of patient strategies (N = 1090)	119 (21.9%)	150 (27.4%)	269 (24.7%)	p = 0.03
**Healthcare system determinants**	Availability of formal healthcare services (N = 1090)	462 (85.1%)	473 (86.5%)	935 (85.8%)	p = 0.5
	Affordability of formal healthcare services (N = 1090)	387 (71.3%)	380 (69.5%)	767 (70.4%)	p = 0.5
**Healthcare utilization**	Place of Treatment* (N = 263)				
	Private healthcare services	36 (43.9%)	70 (38.7%)	106 (40.3%)	p = 0.42
	Public healthcare services	46 (56.1%)	111 (61.3%)	157 (59.7%)	
	Health Care Utilization (N = 1086)				
	Utilization in last 6 months	82 (15.2%)	181 (33.2%)	263 (24.2%)	p = 0.00
	No Utilization in last 6 months	459 (84.8%)	364 (66.8%)	823 (75.8%)	

*Respondents could provide more than one answer. First answer taken.

### Qualitative findings

The key themes emerging from the first stage of qualitative data analysis are summarized by interview respondent in Table 4.

**Table 4:** **Key themes by interview respondent**

A	i) Predictions of future illness
	ii) Resourcefulness in use of treatment or utilizing healthcare from an “unauthorized” source
B	i) Frustration with clinics treating the symptom and not the cause
	ii) Belief that earlier medical neglect caused present condition; Coping with the help of family
C	i) Medical-related stress aggravating chronic condition
	ii) Coping with the help of friends and community based organizations
D	i) Determination required in dealing with formal healthcare services
E	i) Fear of consequences of leaving condition untreated
	ii) Ambivalence towards clinic and lifelong medication
F	i) Acceptance of a flawed healthcare service
G	i) Pragmatic approach to treating chronic illness, using a wide variety of approaches
	ii) Adapting and living with chronic illness
H	i) Fear of side effects of pharmaceutical medication
I	i) Regular use of clinic, but improved health attributed to alternative remedies
J	i) Encouraged by family members with same disease
	ii) Arguments with nurses at the clinic
K	i) View of condition in a non-specific way, as a part of life:
L	i) Invasive encounters with medical system

A number of themes within healthcare narratives begin to explain low healthcare utilzation in Soweto. The first theme emerged in interview B, relating to frustration with government clinics for only treating the symptoms, as opposed to the causes of NCDs. In this case study, the participant, Monica aged 50 described suffering from tuberculosis (TB) since she was a young girl and this has had a significant impact on her spine. She noticed symptoms of arthritis in the wrists and fingers and reported chronic suffering from the disease, although she had never been diagnosed. She rarely went to the clinic because of mistrust and said: “When I go to the clinic what happens is that I sometimes feel pain in my bones, especially my hands and I cannot even hold things properly. But then the sisters at the clinic will normally give me pain killers.” Despite her care-seeking, Monica has yet to receive a diagnosis or formal treatment for arthritis. Notably, with this case study, the role of earlier medical neglect emerged as a contributing factor to mistrust of formal healthcare services.

Additional themes suggesting reasons for avoiding formal healthcare services include the stress of visiting formal healthcare facilities (interviews C and E) and uneasiness with regularly taking medication for NCDs. This last theme is particularly prevalent in Rosaline’s case study, highlighted below (‘Medicine-body interaction’ subsection).

### Medicine-body interaction

In her mid-forties, Rosaline was diagnosed with diabetes and hypertension three years before the interview. Eventually she sought care at a local clinic and now attends the clinic monthly. Rosaline takes diabetic and blood pressure medicine but admittedly doesn’t adhere to her physician’s recommendation: “One tablet, Nordforin, fell on the floor, so I said ‘OK, I’ll take another one.’ So I take that pill and throw it in the sink. I close the tap, and wash the dishes. I finish, take out the water and, I thought it would melt. And it didn’t. And I got worried. ‘What is happening when I drink these tablets every day in my system?”’ In addition to these medicines, Rosaline regularly takes Natural Health Tea to help with blood circulation and high blood pressure, and she takes Jiaogulan tea to delay aging and regulate body fat. “I don’t like drinking these tablets every day. It is not in me, you know. I was talking to my daughter, I said to her: ‘You know, I believe that God is going to heal me, I am not going to drink these pills for the rest of my life.’”

Evidence from the qualitative interviews suggests that women with NCDs also delay attending the clinic upon first noticing the symptoms of NCDs (interviews A, E and H). This delay in utilizing formal healthcare services appears to be largely motivated by fear of the implications of living with NCDs. Ignoring the potential NCD status enables the person to live in a state of false security and comfort. This is expressed in Shirley’s narrative (‘Taking NCDs seriously’ subsection).

### Taking NCDs seriously

Shirley, aged 53, resides in a wealthy part of Soweto. In 2005 she accompanied her son to the clinic and decided to have a check-up. At this time, she was diagnosed with high blood pressure, and three years later with diabetes, cholesterol, and arthritis. She had known for some time that she had high blood pressure but only began to “take it seriously” when her friends and neighbors explained the consequences of leaving it untreated, such as experiencing a stroke. She began taking a wide range of tablets after she was diagnosed, and medication adherence is the focus of her illness experience. She visits the Krugersdorp hospital once a month to pick up her medication and consults with her physician every three months. In the first interview, she described a quick and efficient process. But in the second interview she explained a different clinic experience: “When you are there you become angry. By the time you get there your blood pressure and your sugar level becomes high. When you get there it is as if you are sick because you got angry before you got to the doctor.”

### Quantitative findings

Table 3 relates individual, societal and healthcare system characteristics with NCD status. Overall, one-third (33.2%) of respondents with NCDs reported accessing healthcare services in the last six months compared to only 15.2% of those without NCDs (p < 0.05). Of those who reported accessing some form of healthcare service in the last six months (*n* = 263), almost two-thirds (61.3%) of respondents with NCDs made use of public healthcare services compared to around 56% of respondents without NCDs who also made use of these services, although the difference was not statistically significant. Over two-thirds (69.7%) of respondents with NCDs used prescribed lifelong medication on a daily or regular basis. Respondents reported an average delay of treatment of about 9.6 months (Table 1).

A means comparison test suggests that the age of women with NCDs, was closely, although not significantly, related to healthcare utilization (p = 0.07) (Table 5). Pearson’s chi-square tests indicate that the possession of medical aid was the strongest individual level factor influencing the utilization of healthcare (p = 0.01). Amongst societal level factors, the self-reported use of patient strategies was significantly related to the utilization of healthcare services. Those respondents with NCDs who admitted to using patient strategies when consulting healthcare workers were significantly more likely to utilize healthcare services (p = 0.02). Specific belief in traditional healers, or the belief that there were some conditions which could only be treated by traditional healers, and not by doctors, was closely, although not significantly related to healthcare utilization (p = 0.09), the results showing that such beliefs were positively associated with healthcare utilization. Both availability and affordability of healthcare services were significantly positively related to reported healthcare utilization (p = 0.02 and p = 0.05).

**Table 5:** **Correlating healthcare utilization of persons with NCDs with individual**, **societal and healthcare system determinants**

**Domain**	**Determinants**	**Utilization**	**No utilization**	**Total**	**P-** **value**
**Individual determinants**	Age (mean ± SD) (N = 545)	46 ± 6.7	45.1 ± 6.5	45.7 ± 6.6	p = 0.07
	Socioeconomic Status (N = 542)				
	Low	34 (18.9%)	51 (14.1%)	85 (15.7%)	p = 0.15
	Medium to High	146 (81.1%)	311 (85.9%)	457 (84.3%)	
	Employment (N = 545)				
	Formal or informal paid labor	81 (44.8%)	144 (39.6%)	225 (41.3%)	p = 0.25
	Housewife/ pensioner/ unemployed	100 (55.3%)	220 (60.4%)	320 (58.7%)	
	Medical aid (N = 545)	41 (22.7%)	52 (14.3%)	93 (17.1%)	p = 0.01
	Regular medication (N = 545)	126 (69.6%)	253 (69.5%)	379 (69.5%)	p = 0.98
**Societal determinants**	Shared hlth beliefs family (N = 545)	179 (98.9%)	361 (99.2%)	540 (99.1%)	p = 0.75
	Shared hlth beliefs comm (N = 545)	142 (78.5%)	302 (83%)	444 (81.5%)	p = 0.2
	Specific belief in traditional healers (N = 542)	66 (36.9%)	108 (29.8%)	174 (32.1%)	p = 0.09
	Use of patient strategies (N = 545)	61 (33.7%)	89 (24.5%)	150 (27.5%)	p = 0.02
**Healthcare system determinants**	Availability of formal healthcare services (N = 545)	165 (91.2%)	306 (84.1%)	471 (86.4%)	p = 0.02
	Affordability of formal healthcare services (N = 545)	136 (75.1%)	244 (67%)	380 (69.7%)	p = 0.05

Logistic regression of individual, societal and healthcare system factors influencing the utilization of healthcare services in the last six months by persons with NCDs confirms the importance of the possession of medical aid for utilizing healthcare services (OR = 1.7, CI = 1.01-2.84). It also confirms the close relationship between healthcare utilization and the use of patient strategies for negotiating healthcare access (OR = 1.6, CI = 1.04-2.34) (see Additional file 1).

Additional logistic regression analyses of the influence of individual, societal and healthcare system determinants on type of healthcare service accessed and the likelihood of delaying treatment for persons with NCDs were conducted. The possession of medical aid strongly influenced the use of private as opposed to public healthcare services (OR = 9.6, CI = 3.52-26.11), while the taking of lifelong medication was significantly related to the use of public as opposed to private healthcare services (OR = 0.25, CI = 0.11-0.56). The possession of regular employment was also closely, though not significantly related to the use of private healthcare services (OR = 1.9, CI = 0.89-4.49), most likely due to the fact that medical aid is generally provided through formal employment (see Additional file 1).

Logistic regression analysis of the likelihood of delaying treatment for an NCD suggests that the belief in the superior efficacy of traditional healers over doctors for certain types of diseases is significantly related to the delay of NCD treatment (OR = 2.4, CI = 1.14-4.18). On the other hand, shared healthcare beliefs with the community was significantly related to the absence of delays in treatment for NCDs (OR = 0.32, CI = 0.16-0.64). Closely, although not significantly related to the delay of treatment for NCDs was the professed use of patient strategies when consulting healthcare workers (OR = 1.8, CI = 0.94-3.65) (see Additional file 1).

None of the respondents reported using traditional healers, but the belief in the superior efficacy of traditional healers compared to formal healthcare services was relatively high (31.8%). Most of these responses (19.1%) related to various types of curses, poisonings, or possession, which may be said to lie within the realm of magic or witchcraft (Table 6). Fits, headaches and mental illness were also considered suitable for treatment by traditional healers (17.3%). Around 13.5% and 11.8% believed that traditional healers could treat NCDs and HIV/AIDS, respectively.

**Table 6:** **Belief among people with NCDs that diseases can be treated only by traditional healers** (**n** = **173**)

**Diseases**	**N**	**%**
Witchcraft/curses/poisoning/evil spirit	33	19.1%
Fits/headache/mental illness	30	17.3%
NCDs	23	13.3%
HIV/AIDS	20	11.6%
Unclear	16	9.3%
STI/ sex-related problems	13	7.5%
Ancestral/calling	10	5.8%
Other	10	5.8%
Swollen feet/legs	9	5.2%
Rash/skin disease	5	2.9%
Cleansing/protection/ritual	3	1.7%
TB	1	0.6%
**Total**	**173**	**100**%

## Discussion

This study examines perceptions and behaviors for health-seeking and NCD-care in urban South Africa. Combining qualitative with quantitative data, we found that the relatively low levels of healthcare utilization amongst women with NCDs in Soweto is due in part to low rates of expectation for consistent and high-quality care and a potential mistrust of the medical system (as demonstrated in Rosaline and Shirley’s stories).

Although low utilization of healthcare services has been documented [6,13], our quantitative data underscore the potential severity of low healthcare utilization in Soweto, as only one-third (33.2%) of women with NCDs reported utilizing formal healthcare services. This is alarming as it may indicate poor medical management of NCDs, which may not only exacerbate the condition but also lead to premature mortality.

The case studies suggest that mistrust of formal healthcare services is a potential reason for low healthcare utilization based on past negative experiences, as with Monica (who felt that clinics provided only pain killers rather than diagnosis and treatment) and Shirley (who described a frustrating encounter with bureaucracy at the clinic), or on uncertainty, as with Rosaline (who was apprehensive about the effect of pharmaceutical medication on the body). Across all interviews, themes emerged that indicated an ambivalent relationship between the healthcare user and formal healthcare systems, including pharmaceutical medication. In some cases this manifested in multiple modes of treatment for NCDs. Other studies in the African context suggest that negative experiences of public healthcare services are frequently due to the fact that they are overburdened, resulting in shortage of medication and long waiting times to access care [15].

In our analysis the possession of medical aid was the strongest individual-level factor influencing the use of formal healthcare services. This suggests that low levels of healthcare utilization may be explained in part by economic insecurity, a notion confirmed by our finding that the perceived affordability of formal healthcare services was significantly associated with healthcare utilization.

It is easy to see why the possession of medical aid would be positively related to healthcare utilization, but it is less easy to see why the perceived affordability of healthcare services should prove a factor, especially given the fact that basic healthcare services are free of charge in South Africa. It has been suggested elsewhere that cost-related barriers to healthcare utilization may be associated with costs of transport and medication and complex treatment patterns [5,9]. Our quantitative findings underscore the importance of further exploring how economic insecurity functions as a barrier to healthcare seeking in the urban South African context.

The self-reported use of patient strategies was the strongest societal-level factor associated with healthcare utilization by women with NCDs. Respondents who reported utilizing formal healthcare services were more likely to admit to the use of patient strategies. We classify the use of patient strategies as a societal-level determinant of healthcare utilization with the understanding that the use of patient strategies functions as a norm of social interaction that indicates the development of tacit knowledge guiding interpersonal interactions in the healthcare setting. Werner and Malterud have found in their qualitative study that women with chronic illness have to “work” to ensure that they are perceived as credible patients [16]. But the fact that such behavior is related to increased healthcare utilization suggests that experience of accessing healthcare at public healthcare facilities, combined with resilience, accounts for continuing healthcare use.

Should the lack of trust of formal healthcare services be a key factor in explaining healthcare utilization patterns, then it would be a factor spanning societal and healthcare system factors. Elsewhere it has been suggested that race or ethnicity may contribute to increased mistrust of healthcare services [17], but in the South African context it has been found that poor experiences of public healthcare facilities related to the lack of prompt attention and communication may be of greater concern [18]. Our case studies reveal that many women depend upon alternative and complementary medicine and therapy to complement their ongoing interaction with formal healthcare services. This suggests that self-diagnosis and reliance on traditional medicine emerge as responses to mistrust which simultaneously allow for the exercise of control over the intake of biomedicine.

The quantitative findings indicate that few people trust traditional healers for NCD treatment: only one in ten people interviewed believed a traditional healer could heal their NCD. Those diseases that people believed were treatable by traditional healers, as opposed to doctors, were illnesses of the social, spiritual, and emotional world. At the same time, the general belief in the efficacy of traditional healers over that of formal healthcare workers was associated with a delay in accepting formal long term treatment for NCDs. Other studies in the South African context have shown that the use of traditional healers and other alternative therapies is generally associated with poor adherence to formal treatment regimens [19,20]. Our findings suggest that the belief in the efficacy of traditional healers may delay the onset of treatment at a formal healthcare facility, but that once such contact is initiated, the enduring belief in traditional healers does not affect the ongoing utilization of formal healthcare services, suggesting that such experiences may modify the belief in traditional healers, confining their utility to a more social role.

The availability and affordability of healthcare services emerged as significant healthcare system factors influencing the utilization of healthcare services, although they did not emerge as significant factors in the multi-factorial model. Nor did these emerge as themes in our qualitative analysis. Our results therefore indicate that while initial gains made in improving healthcare access in Soweto have been significant, further work is needed particularly in enhancing the experience of the healthcare visit.

The cross-sectional study design did not allow us to investigate healthcare utilization trends over time in relation to independent variables, such as socio-economic status and education. Future studies should also include surveys of healthcare providers within different areas of Soweto, and should relate these findings with healthcare users’ beliefs and practices. There is a need to include role-players from government and community sectors in the research process, and to ensure that the findings of future research feed into healthcare strengthening programs. Nevertheless, this research underscores the need for more research in urban South Africa on people’s beliefs about and access to healthcare for NCDs.

## Conclusion

Our study has highlighted the role played by poor experiences of formal healthcare services and low levels of trust in explaining low healthcare utilization in Soweto. Demand-side reforms, such as increasing the number of healthcare workers and medical supplies will certainly prove important in changing the general perception of healthcare services and increasing healthcare utilization. At the same time, our findings underscore the importance of previous healthcare experiences and community perceptions of healthcare in shaping individuals’ choice to utilize formal healthcare services. Low healthcare utilization in Soweto has as much to do with poor trust of formal healthcare services, based on past negative experiences, as it does with lack of information and misconceptions about NCDs.

Trust, understood as the relational commitment between individuals in social settings, is developed along with the development of the institutional frameworks for communication and decision-making as well as the personal characteristics of the individuals involved in the trusting relationship, such as competence, empathy, congeniality and fairness [21,22]. As such, it proves the linking concept between demand-side and supply-side healthcare reforms required to improve the quality of the healthcare interaction, and consequently the utilization of healthcare services for the management of NCDs. Responding to the evident lack of trust in formal, particularly public healthcare systems in Soweto, and in South Africa, should be based on high trust management practices by healthcare service providers.

### Consent

Written informed consent was obtained from all research participants for the publication of this report.

## Additional file

Additional file 1:
**Multivariate logistic regression of individual, societal and healthcare system determinants on healthcare utilization, type of healthcare utilization and self-reported delay of formal healthcare treatment for NCDs.**
