# Supplementary material for: A mixed methods exploration of patterns of healthcare utilization of urban women with non-communicable disease in South Africa
Source: BMC Health Serv Res. 2014 Nov 4;14:528. doi: 10.1186/s12913-014-0528-y (PMC4231186; doi:10.1186/s12913-014-0528-y)
Supplement: Additional file 1: — Multivariate logistic regression of individual, societal and healthcare system determinants on healthcare utilization, type of healthcare utilization and self-reported delay of formal healthcare treatment for NCDs. [file 12913_2014_528_MOESM1_ESM.doc]

Additional file1: Multivariate logistic regression of individual, societal and healthcare system determinants on healthcare utilization, type of healthcare utilization and self-reporte delay of formal healthcare treatment for NCDs

| **Domain** | **Determinants** | **Healthcare utilization (n=539)** | | | **Private healthcare utilization (n=176)** | | | **Delay in health seeking (n=408)** | | |
| --- | --- | --- | --- | --- | --- | --- | --- | --- | --- | --- |
|  |  | **OR** | **95% CI** | **p-value** | **OR** | **95% CI** | **p-value** | **OR** | **95% CI** | p-value |
| **Individual Determinants** | Medical aid | 1.7 | (1.02-2.84) | 0.04 | 9.59 | (3.52-26.11) | 0 | 1.3 | (0.47-3.57) | 0.61 |
|  | Age | 0.98 | (0.95-1.01) | 0.21 | 1.03 | (0.97-1.09) | 0.37 | 1.02 | (0.97-1.07) | 0.42 |
|  | Employment | 1 | (0.66-1.5) | 0.98 | 2 | (0.89-4.49) | 0.1 | 0.58 | (0.26-1.26) | 0.17 |
|  | Socioeconomic status | 0.66 | (0.4-1.09) | 0.11 | 1.43 | (0.51-4.03) | 0.49 | 1.39 | (0.5-3.81) | 0.53 |
|  | Presence of NCD and regular meds | 1.12 | (0.74-1.69) | 0.59 | 0.25 | (0.11-0.56) | 0 | 1.45 | (0.64-3.26) | 0.37 |
| **Societal Level Determinants** | Specific belief in traditional healers | 1.34 | (0.91-1.98) | 0.14 | 0.98 | (0.47-2.08) | 0.97 | 2.18 | (1.14-4.18) | 0.02 |
|  | Shared healthcare beliefs with community | 0.82 | (0.51-1.32) | 0.42 | 1.38 | (0.54-3.52) | 0.5 | 0.32 | (0.16-0.64) | 0 |
|  | Use of patient strategies | 1.56 | (1.04-2.34) | 0.03 | 0.86 | (0.39-1.89) | 0.7 | 1.85 | (0.94-3.65) | 0.08 |
| **Healthcare System Determinants** | Availability of formal healthcare services | 1.68 | (0.81-3.49) | 0.16 | 0.52 | (0.12-2.31) | 0.39 | 2.47 | (0.71-8.65) | 0.16 |
|  | Affordability of formal healthcare services | 1.12 | (0.68-1.85) | 0.67 | 0.89 | (0.34-2.38) | 0.82 | 0.55 | (0.25-1.21) | 0.14 |
